# Factors associated with reporting of abuse against children and adolescents by nurses within Primary Health Care^1^

**DOI:** 10.1590/0104-1169.0050.2515

**Published:** 2014

**Authors:** Ana Carine Arruda Rolim, Gracyelle Alves Remigio Moreira, Sarah Maria Mendes Gondim, Soraya da Silva Paz, Luiza Jane Eyre de Souza Vieira

**Affiliations:** 2 Doctoral student, Faculdade de Ciências Médicas, Universidade Estadual de Campinas, Campinas, SP, Brazil; 3 Doctoral student, Programa de Pós-graduação em Saúde Coletiva, Associação Ampla - Universidade Estadual do Ceará, Fortaleza, CE, Brazil, Universidade Federal do Ceará, Fortaleza, CE, Brazil and Universidade de Fortaleza, Fortaleza, CE, Brazil; 4 RN, student of the Specialization course in Occupational Nursing, Universidade Estadual do Ceará, Fortaleza, CE, Brazil; 5 RN, student of the Specialization course in Management and Audit of Systems and Health Services, Faculdade Leão Sampaio, Fortaleza, CE, Brazil; 6 PhD, Full Professor, Universidade de Fortaleza, Fortaleza, CE, Brazil

**Keywords:** Mandatory Reporting, Violence, Child, Adolescent, Primary Health Care

## Abstract

**OBJECTIVE:**

to analyze the factors associated with the underreporting on the part of nurses within Primary Health Care of abuse against children and adolescents.

**METHOD:**

cross-sectional study with 616 nurses. A questionnaire addressed socio-demographic data, profession, instrumentation and knowledge on the topic, identification and reporting of abuse cases. Bivariate and multivariate logistic regression was used.

**RESULTS:**

female nurses, aged between 21 and 32 years old, not married, with five or more years since graduation, with graduate studies, and working for five or more years in PHC predominated. The final regression model showed that factors such as working for five or more years, having a reporting form within the PHC unit, and believing that reporting within Primary Health Care is an advantage, facilitate reporting.

**CONCLUSION:**

the study's results may, in addition to sensitizing nurses, support management professionals in establishing strategies intended to produce compliance with reporting as a legal device that ensures the rights of children and adolescents.

## Introduction

Acknowledged worldwide as a social and public health problem due to its impact on the morbidity and mortality of the population and also on the routine of human experiences^(^ 1 ^)^, violence is deeply rooted in social, economic and political structures, as well as in individual consciousness and cultural dynamics. Violence against children and adolescents entails a breach of the duty on the part of adults and society, in general, to protect these individuals, as well as a trivialization of the rights of children and adolescents to be treated as subjects and people under special conditions of growth and development.

The magnitude of this problem can be found in studies that show violence against children and adolescents to be the main causes of death and disease among these populations in many countries^(^ 2 ^-^ 4 ^)^, including Brazil^(^ 5 ^)^. The magnitude of this problem in the international scenario concerns governments, researchers and civil society, because its repercussions for the future lives of this members of these groups are significant^(^ 2 ^,^ 6 ^)^.

In the Brazilian context, as part of the strategy used to cope with this problem, the Child and Adolescent Statute (ECA) has established that healthcare workers and those in the field of education are supposed to report maltreatment against children and adolescents^(^ 7 ^)^. In the health field, this practice is supported by Decree No. 1.968/2001, which institutionalized mandatory reporting of abuse against children and adolescents who receive care from the Unified Health System (SUS)^(^ 8 ^)^ and also by Decree No. 104/2011, which establishes that domestic violence, sexual and/or other types of violence is the 45^th^ event in a list of mandatory reporting^(^ 9 ^)^.

As part of the Brazilian healthcare model, Primary Health Care (PHC) presents a privileged opportunity to identify and manage situations of abuse perpetrated against children and adolescents. The reason is that this model is intended to prevent disease and harm and is grounded on health promotion to encourage coping with violence against this population.

In this field of collective health, nurses stand out because they have academic background that qualify them to perform actions that promote health as well as family care^(^ 10 ^)^. This profession has incorporated new practices that transcend the technical-healing model due to the complex demands presented within PHC^(^ 11 ^)^. In this context, maltreatment of children and adolescents and its consequences reverberate in the routines of family strategy health teams and demand from nurses and other healthcare workers an ethical and legal response in accordance with the precepts that govern this topic. 

Nonetheless, studies indicate that PHC-unit-originating reports of violence are a challenge for many reasons including lack of preparation and poor management of cases, in addition to a fear of personal or professional retaliation^(^ 11 ^-^ 12 ^)^. From this perspective, this study is relevant because it lists the factors that facilitate reporting of abuse against children and adolescents in regard to the professional and citizenship roles of nurses working in PHC, an aspect seldom addressed in the literature. 

We expect that these results will support the planning of actions to facilitate the reorientation of practices involving management and care provided by PHC units so that reporting becomes effective. Hence, the objective was to analyze the factors associated with juvenile abuse reporting by nurses working in PHC.

## Method

A cohort cross-sectional analysis was performed using the database of a larger study titled "Violence involving children and adolescents: conditioning factors, reporting processes and coping mechanisms", the objective of which was to analyze the reporting, on the part of healthcare workers (physicians, nurses, and dentists) from the Health Family team, of abuse committed against children and adolescents in cities in Ceará, Brazil. This paper specifically elaborates on reporting on the part of nurses. 

**Figure 1 -:**
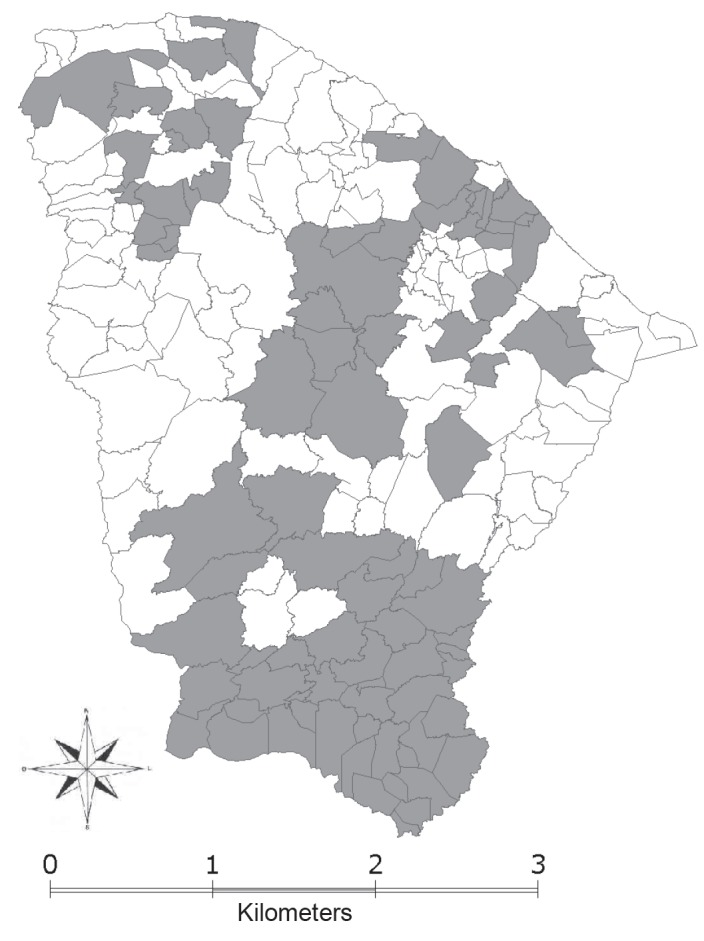
Map of the state of Ceará, Brazil showing the cities under study

The state of Ceará is composed of 184 cities. This study included the investigation of 46.2% of those cities, i.e., 85 cities, spread out in all the health regions within the state (Figure 1).

The population of nurses was obtained based on data provided by the Primary Care Department (PHD), which at the time of the study, listed 1,014 nurses; 616 (60.7%) of these completed the survey.

Data were collected between 2010 and 2012. All the nurses working in PHC units in the participant cities received letters inviting them to participate in the study, free and informed consent forms to sign and submit, and the questionnaire. These papers, previously organized in sealed envelopes identified by city, were sent to the managers of the Regional Health Coordinators and/or the City Health Departments, which became responsible for sending the envelopes to the nurses in each city. The completed forms were returned following the inverse flow. 

The structured questionnaire was composed of 32 adapted and revised questions with the following analytical domains: socio-demographic data, professional background, instrumentation and knowledge regarding the topic, identification and reporting of maltreatment against children and adolescents.

The study's outcome was the reporting of abuse against children and adolescents and the predictor variables included: sex; age; marital status; time since graduation; graduate studies; time working in the PHC unit; and whether training was received; being aware of ECA; being aware of the reporting form; being aware of the reporting form within the PHC unit; trust in protection agencies; knowing where to refer cases; fearing legal involvement; reading about the subject; discussing the subject at work; knowing assistance was provided to victims; and believing that the implementation of reporting within PHC was an advantage.

The Chi-square test (χ^2)^ was used to analyze potential association between the outcome and the predictor variables. P<0.05 was established to determine statistical significance. Multiple logistic regression was used, in which the predictor variables that showed association with the outcome with a significance of p<0.25 were included. The variables with a level of significance p<0.05 remained in the multiple model. The strength of association between the outcome and the predictor variables was expressed in raw and adjusted Odds Ratio (OR), with a Confidence Interval (CI) of 95%. All the questionnaires were checked and entered into the database through double entry to verify consistency of data, which were input into the SPSS (SPSS Inc., Chicago, United States), version 17.0. STATA was used for the analysis (Stata Corp LP, College Station, TX 77845, USA), version 11.0.

The study was approved by the Institutional Review Board at the University of Fortaleza - UNIFOR (referee report No. 072/2007).

## Results

The nurses were 32.5 years old (SD±7.6) on average. The following profile predominated: women (86.4%); from 21 to 32 years old (60.1%); not married (51.7%); with five or more years since graduation (59.2%); with graduate studies (83.1%); and five or more years working within PHC (52.4%).

In regard to the identification of abuse against children and adolescents in their professional practice, 56.9% of the nurses reported they had not identified cases of abuse, while 43.1% affirmed they had. Of these, 69.7% identified the abuse situations through the reports of the victims themselves, family members, or others. In regard to the reporting of identified cases, 58.4% did not file a report and 41.6% did report the abuse.

The variables of time since graduation (p=0.004) and time working within PHC (p=0.004) were statistically associated with the reporting of abuse against children and adolescents, while sex, age, marital status and graduate studies presented p>0.05 (Table 1).

**Table 1 -:** Bivariate analysis of abuse reporting and socio-demographic data and professional background of nurses. Primary Health Care, CE, Brazil, 2010-2012

Variable		Nurse reported maltreatment						Not adjusted	
		Yes			No				
		n	%		n	%		OR (CI95%)	p
Sex (n=317)								1.10 (0.54–2.28)	0.763
	Male	17	12.9		26	14.1			
	Female	115	87.1		159	85.9			
Age (in years) (n=282)								1.00 (0.60–1.66)	0.979
	21 – 32	64	54.7		90	54.5			
	> 32	53	45.3		75	45.5			
Marital status (n=317)								1.05 (0.65–1.69)	0.811
	Married	71	53.8		97	52.4			
	Not married	61	46.2		88	47.6			
Time since graduation (n=317)								2.06 (1.21–3.55)	0.004
	< 5 years	30	22.7		70	37.8			
	≥ 5 years	102	77.3		115	62.2			
Graduate studies (n=315)								1.56 (0.73–3.43)	0.212
	Yes	118	90.1		157	85.3			
	No	13	9.9		27	14.7			
Time working within PHC* (n=316)								1.99 (1.20–1.30)	0.004
	< 5 years	38	71.0		83	44.9			
	≥ 5 years	93	29.0		102	55.1			

* Primary Health Care

The variables of received training in the subject, is familiar with the reporting form, with the reporting form at the PHC unit, trusts in protection agencies, knows where to refer cases, fears legal involvement, reads about the subject, and believes that implementing reporting in PHC units is an advantage, were statistically associated with the outcome (p<0.05). The remaining variables did not present statistically significant differences (Table 2).

**Table 2 -:** Bivariate analysis between abuse reporting and instrumentation and knowledge of nurses. Primary Health Care, CE, Brazil, 2010-2012

Variable		Nurse reported abuse						Not adjusted	
		Yes			No				
		n	%		n	%		OR (IC95%)	p
Received training (n=313)								2.46 (1.49–4.06)	<0.001
	Yes	64	48.5		50	27.6			
	No	68	51.5		131	72.4			
Is familiar with ECA* (n=315)								1.60 (0.81–3.26)	0.146
	Yes	114	87.7		151	81.6			
	No	16	12.3		34	18.4			
Is familiar with the reporting form (n=315)								3.96 (2.28–6.98)	<0.001
	Yes	107	81.1		95	51.9			
	No	25	18.9		88	48.1			
Is familiar with the reporting form from the PHC unit^†^ (n=307)								3.47 (2.10–5.75)	<0.001
	Yes	79	62.7		59	32.6			
	No	47	37.3		122	67.4			
Trusts in the agencies of protection (n=310)								1.67 (1.02–2.76)	0.030
	Yes	86	67.2		100	54.9			
	No	42	32.8		82	45.1			
Knows the proper place to refer cases (n=311)								4.17 (2.11–8.71)	<0.001
	Yes	116	89.9		124	68.1			
	No	13	10.1		58	31.9			
Fears legal involvement (n=309)								1.57 (0.97–2.55)	0.049
	Yes	61	48.0		108	59.3			
	No	66	52.0		74	40.7			
Reads about the subject (n=317)								1.61 (0.98–2.63)	0.042
	Yes	56	42.4		58	31.3			
	No	76	57.6		127	68.7			
The subject is discussed at work (n=317)								1.42 (0.88–2.28)	0.121
	Yes	73	55.3		86	46.5			
	No	59	44.7		99	53.5			
Knows assistance is provided to victims (n=311)								1.20 (0.68–2.10)	0.491
	Yes	33	25.8		41	22.4			
	No	95	74.2		142	77.6			
Believes reporting within PHC is an advantage^‡^ (n=314)								2.40 (1.08–5.73)	0.019
	Yes	122	92.4		152	83.5			
	No	10	7.6		30	16.5			

* Child and Adolescent Statute

‡ Primary Health Care

The variables selected (p<0.25) for the multiple analysis included: time since graduation; graduate studies; time working within PHC; received training; is familiar with ECA; is familiar with the reporting form; is familiar with the reporting form from the PHC unit; trusts protection agencies; knows where to refer cases; fears legal involvement; reads about the subject; the subject is discussed at work; and believes it is an advantage to implement reporting within PHC.

Table 3 presents the results of the logistic regression. Time working within PHC, reporting form from the PHC unit, knows where to refer cases, fears legal involvement, and believes that implementing reporting within PHC is an advantage, remained associated with the outcome in the final logistic regression model.

**Table 3 -:** Multivariate analysis of abuse reporting and associated factors. Primary Health Care, CE, Brazil, 2010-2012

Variable		Nurse reported abuse						Adjusted	
		Yes			No				
		N	%		n	%		OR (IC95%)	p
Time working within PHC* (n=316)								3.09 (1.74–5.49)	<0.001
	< 5 years	38	71.0		83	44.9			
	≥ 5 years	93	29.0		102	55.1			
Reporting form from the PHC unit^†^ (n=307)								3.73 (2.18–6.38)	<0.001
	Yes	79	62.7		59	32.6			
	No	47	37.3		122	67.4			
Knows where to refer cases (n=311)								3.33 (1.60–6.93)	0.001
	Yes	116	89.9		124	68.1			
	No	13	10.1		58	31.9			
Fears legal involvement (n=309)								1.87 (1.09–3.20)	0.021
	Yes	61	48.0		108	59.3			
	No	66	52.0		74	40.7			
Believes that implementing reporting within PHC is an advantage* (n=314)								2.83 (1.21–6.63)	0.016
	Yes	122	92.4		152	83.5			
	No	10	7.6		30	16.5			

* Primary Health Care

† Primary Health Care Unit

Those working within PHC for five or more years were 3.09 times more likely to report abuse. Having a reporting form for within the PHC unit increased the likelihood of filling a report by three times. Likewise, knowing where to refer cases of child abuse increased by 3.33 times the reporting practice. Not being afraid of legal involvement almost doubled the likelihood of reporting. Finally, believing that abuse reporting within PHC is an advantage increased the likelihood of complying with devices that regulate reporting by almost three times (Table 3). 

## Discussion

Even though this study reports factors associated with the reporting of abuse among PHC nurses, data show that underreporting predominates, even when nurses do identify cases of abuse against children and adolescents. The organization of the work process within the context of PHC to meet social demands is not conducive to meeting the political guidelines and principles intended to reorient the healthcare model.

This weakness regarding reporting of abuse in the practice of nurses is also verified in other Brazilian regions^(^ 11 ^,^ 13 ^)^ and in countries from other socio-cultural contexts^(^ 14 ^-^ 16 ^)^. One study conducted in Israel with 143 nurses and 42 physicians revealed that 60.0% of the professionals did not report abuse^(^ 16 ^)^. Even though more advanced systems have been established in the USA for the reporting of child abuse for a longer period of time, there still are barriers that hinder nurses from filing abuse reports^(^ 15 ^)^.

One potential explanation for underreporting is the misinterpretation of the term "reporting", because, in Brazil, it is culturally and historically associated with denouncement^(^ 17 ^)^. In this sense, considering the role healthcare workers within PHC play in regard to the community and area covered, according to the logic of the Brazilian health model, nurses may be choosing timid behavior in regard to sensitive and complex issues, as is the case of violence. This represents an obstacle because underreporting prevents health management from completely recognizing the magnitude of the problem; health managers depend on information at the local level to implement effective strategies.

Association between child and adolescent abuse reporting and longer time working within PHC, as revealed in the analysis, shows that workers with greater professional experience feel better prepared to deal with this problem. One hypothesis is that the professional who develops activities within PHC over longer periods of time may have had more opportunities to witness situations of violence, and therefore, may be more familiar with the proper management of such cases. The importance of having greater experience in the service is shown in a study^(^ 18 ^)^ reporting that "daily contact with violence perpetrated against children awakens in the professional a state of alertness, which mobilizes him/her to identify signs indicating violence."

Another hypothesis is that nurses may have improved the way they deal with situations of child abuse, including the decision to report it, for having become more professionally mature and having received training on the subject. The State Health Department in Ceará, Brazil has promoted systematic training on coping with violence, focusing on the reorientation of the practice of PHC workers. Some nurses composing the study sample may have received such training, which would explain the increased likelihood of reporting observed here.

Having reporting forms available at the PHC unit is also associated with higher levels of reporting among nurses. Other studies have shown that the existence of protocols establishing conduct within the health unit provides tools for professionals to be more active, even if what is available is not the reporting form, per se; other forms to communicate violence to competent authorities may exist^(^ 19 ^)^.

Therefore, management should at least ensure the workers have the material necessary for qualified practice within PHC. Complaints of workers and patients regarding the inadequacy of the physical structure and insufficiency of supplies in health units are frequently reported by the Brazilian media and confirmed in the literature^(^ 20 ^-^ 21 ^)^. These complaints confirm that problem-solving actions intended to meet these demands should be a priority; otherwise, there is a risk of compromising the flow of care delivery and hindering procedures that would enable solutions recommended by the law.

Being aware of where cases should be referred also increases the likelihood of reporting. In some sense, it may reflect the commitment of nurses to the integral health of children and adolescents in situations of violence and their confidence in agencies that provide support and protection. Studies highlight the nurse as an important professional in the management of cases of abuse within PHC, especially when compared to other professionals^(^ 11 ^-^ 12 ^)^.

Nonetheless, would nurses "cease" their co-participation in following up the cases after referring situations of violence to competent authorities and experiencing a feeling of 'mission accomplished' (in accordance with the law)? Does it mean that other professionals within the support and protection network will monitor the cases? Because the conditions of violence have not yet been internalized in the health-work process; professionals often do not feel prepared and competent to face the problem. One study^(^ 11 ^)^ shows reports of PHC nurses who believe that child and adolescent abuse is within the sphere of other professionals (e.g., social workers or psychologists).

The fact that nurses do not fear becoming legally involved also encouraged the reporting of abuse. This information confirms that having a protection and support network for those suffering violence, as well as professionals who become responsible for the reporting, is essential. The establishment and operationalization of this network is the role of managers and involves the three governmental spheres. This network should be linked to other social segments, while the support provided by the Public Prosecutor in holding perpetrators accountable is key to minimizing personal and professional reprisal.

Another investigation reveals that PHC workers face a real dilemma concerning the decision to report abuse, even when they are aware of their legal obligations, and may opt for a "friendly neighbor policy" in order to ensure their own safety within the work environment when exposed to situations that put their physical or moral integrity at risk^ (11)^.

Another relevant aspect is that nurses who believe that reporting within PHC is an advantage are more inclined to filing reports. Perhaps, in accordance with the socio-sanitary considerations, these workers acknowledge the importance of ties established with the living territory and regard the nuclear family as the driving force in the recovery of values. They also perceive violence as a problem in the health sector, acknowledging the concept of multidimensional health addressed in the Brazilian Constitution^(^ 22 ^)^. Another relevant aspect is that nurses who believe that implementing reporting within PHC are more inclined to file reports.

After presenting these interpretations and arguing for their validity, it is important to note the study's limitations. A total of 60.7% of the nurses consented to participate in the study, which may imply that the ones who adhered to the study are more committed to health actions, more familiar with the topic, and/or received prior training. Despite these considerations, which would explain the significant percentage of reporting observed, there are weaknesses in the identification and reporting of child and adolescent abuse. Additionally, even though this study presents an analysis based on primary data, generalizations within the nursing profession are not possible, though the data presented here corroborates the literature portraying other levels of healthcare.

## Conclusion

This study clarified aspects related to reporting of abuse against children and adolescents by nurses within PHC. The regression logistic model showed that factors such as working within PHC for five or more years, having a reporting form within the PHC unit, knowing where to refer cases, not being afraid of legal involvement, and believing that reporting within PHC is advantageous, favor complying with this legal device that ensures the rights of this population.

We believe these results will sensitize nurses and also be used by management professionals to guide strategies for effective reporting. This study reveals the need to include continuous education processes within the PHC services to encourage, both professionals delivering care and those in management functions, to reflect upon this issue and identify intervenient factors that perpetuate underreporting of violence perpetrated against children and adolescents and, at the same time, weaken the system that ensure the rights of this population.

## References

[1] Lima MADS, Rückert TR, Santos JLG, Colomé ICS, Costa AM (2009). Atendimento aos usuários em situação de violência:
concepções dos profissionais de unidades básicas de saúde. Rev Gaúcha Enferm..

[2] Troiano MA (2011). Child Abuse. Nurs Clin North Am..

[3] Liao M, Lee AS, Roberts-Lewis AC, Hong JS, Jiao K (2011). Child maltreatment in China: An ecological review of the
literature. Child Youth Serv Rev..

[4] Finkelhor D, Turner H, Ormrod R, Hamby SL (2013). Violence, crime, and abuse exposure in a national sample
of children and youth: an update. JAMA Pediatr..

[5] Matos KF, Martins CBG (2013). Mortalidade por causas externas em crianças,
adolescentes e jovens: uma revisão bibliográfica. Rev Espaço para a Saúde..

[6] Fenton MC, Geier T, Keyes K, Skodol AE, Grant BF, Hasin DS (2013). Combined role of childhood maltreatment, family history,
and gender in the risk for alcohol dependence. Psychol Med..

[7] (1990). Lei 8.069, de 13 de julho de 1990 (BR). [Internet]. Dispõe
sobre o Estatuto da Criança e do Adolescente e dá outras providências.

[8] (2001). Portaria GM/MS n. 1.968, de 25 de outubro de 2001 (BR).
[Internet]. Dispõe sobre a notificação, às autoridades competentes, de casos de
suspeita ou de confirmação de maus-tratos contra crianças e adolescentes atendidos
nas entidades do Sistema Único de Saúde.

[9] (2011). Portaria n. 104, de 25 de janeiro de 2011 (BR). [Internet].
Define as terminologias adotadas em legislação nacional, a relação de doenças,
agravos e eventos em saúde pública de notificação compulsória em todo território
nacional e estabelece fluxos, critérios, responsabilidades e atribuições aos
profissionais de saúde..

[10] Oliveira RG, Marcon SS (2007). Trabalhar com famílias no Programa de Saúde da Família:
a prática do enfermeiro em Maringá-Paraná. Rev Esc Enferm USP..

[11] Aragão AS, Ferriani MGC, Vendruscollo TS, Souza SL, Gomes R (2013). Primary care nurses' approach to cases of violence
against children. Rev. Latino-Am. Enfermagem..

[12] Lima MCCS, Costa MCOC, Bigras M, Santana MAO, Alves TDB, Nascimento OC (2011). Atuação profissional da atenção básica de saúde face à
identificação e notificação da violência infanto-juvenil. Rev Baiana de Saúde Pública..

[13] Silva MAI, Ferriani MGC (2007). Domestic violence: from the visible to the
invisible. Rev. Latino-Am. Enfermagem..

[14] Eisbach SS, Driessnack M (2010). Am I sure I want to go down this road? Hesitations in
the reporting of child maltreatment by nurses. J Spec Pediatr Nurs..

[15] Smith JS, Rainey SL, Smith KR, Alamares C, Grogg D (2008). Barriers to the mandatory reporting of domestic violence
encountered by nursing professionals. J Trauma Nurs..

[16] Natan MB, Faour C, Naamhah S, Grinberg K, Klein-Kremer A (2012). Factors affecting medical and nursing staff reporting of
child abuse. Int Nurs Rev..

[17] Dobke VM, Santos SS, Dell'Aglio DD (2010). Abuso sexual intrafamiliar: da notificação ao depoimento
no contexto processual-penal. Temas Psicol..

[18] Angelo M, Prado SI, Cruz AC, Ribeiro MO (2013). Vivências de enfermeiros no cuidado de crianças vítimas
de violência intrafamiliar: uma análise fenomenológica. Texto Contexto Enferm..

[19] Deslandes S, Mendes CHF, Lima JS, Campos DS (2011). Indicadores das ações municipais para a notificação e o
registro de casos de violência intrafamiliar e exploração sexual de crianças e
adolescentes. Cad Saúde Pública..

[20] Conill EM (2008). Ensaio histórico-conceitual sobre a Atenção Primária à
Saúde: desafios para a organização de serviços básicos e da Estratégia Saúde da
Família em centros urbanos no Brasil. Cad Saúde Pública..

[21] Senna MCM, Costa AM, Silva LN (2010). Atenção à saúde em grandes centros urbanos: desafios à
consolidação do SUS. Soc Debate..

[22] Constituição (1988) (BR) (1988). Constituição da República Federativa do Brasil.

